# Effect of combined density gradient centrifugation on X- and Y- sperm separation and chromatin integrity

**Published:** 2012-09

**Authors:** Tahereh Esmaeilpour, Leila Elyasi, Soghra Bahmanpour, Alireza Ghannadi, Ahmad Monabbati, Farzaneh Dehghani, Marjaneh Kazerooni

**Affiliations:** 1 *Department of Anatomical Sciences, *Medical School, *Shiraz University of Medical Sciences, Shiraz, Iran.*; 2 *Islamic Azad University, Larestan, Iran.*; 3 *Department of Pathology, Shiraz University of Medical Sciences, Shiraz, Iran.*; 4 *Shiraz Infertility Center, Shiraz, Iran.*

**Keywords:** *Albumin gradient*, *Double-labeled fluorescence in situ hybridization*, *Sperm separation*, *Sex ratio*, *Sperm chromatin*

## Abstract

**Background:** It has been claimed that by using different washing methods, the sperms can be separated according to size, motility, density, chromosomal content and surface markings and charge. These methods also reduce sperm chromatin deficiencies and screen the sperms before applying in assisted reproduction techniques.

**Objective: **This study compared simple density gradient methods and a combined method with albumin density gradient and PureSperm separation (alb/PureSperm) for sex preselection by double fluorescence in situ hybridization (FISH) versus chromomycin A3 staining to determine chromatin integrity.

**Materials and Methods:** 30 normal semen samples were prepared with PureSperm, albumin gradients and alb/PureSperm. All samples were then stained by FISH and chromomycin A3. The results were compared with SPSS 11.5 and the Kruskal-Wallis test.

**Results:** The proportion of X-bearing spermatozoa by PureSperm separation (47.58±5.67) and Y-bearing spermatozoa by albumin gradient (46.13±3.83) methods were slightly higher than in putative normal sperm samples (1:1), but there were no significant differences in the X- or Y- bearing spermatozoa counts among the three methods. Albumin gradient separation tended to underestimate abnormal spermatozoa compared to PureSperm and combined alb/PureSperm.

**Conclusion:** Routine separation methods slightly enriched X- or Y- bearing spermatozoa, but the differences were not significant for clinical purposes. The combined alb/PureSperm method had no advantages for assessing sex ratio or chromatin integrity compared to simpler gradient methods.

## Introduction

The desire to control the sex of one's offspring has its roots in ancient cultures. It was believed that mono-orchydectomy, the position and timing of sexual intercourse and diet affected the baby's sex (1, 2). Gender selection has been used to minimize sex-linked genetic diseases and for family gender balancing (3). Many investigators have assessed enrichment methods to alter the sex ratio of human spermatozoa. Among the techniques tried to date are albumin gradient separation, the swim-up procedure and percoll gradient separation most of which have yielded inconsistent results (4-6). 

Some researchers have reported success in enriching Y-bearing spermatozoa, whereas others have recommended enriching X- bearing spermatozoa and still other studies reported no success with enrichment (4, 7, 8). Part of this controversy might be due to the use of quinacrine staining and single-labeled fluorescence in situ hybridization (FISH) methods, which published evidence has shown to be unreliable (6, 9). 

Cell and molecular biology provide various methods for assessing the proportion of X- and Y-bearing spermatozoa by reliable procedures such as PCR, reanalysis of flow cytometry-sorted sperm samples for DNA content and FISH. Double-labeled FISH provides a more accurate and reliable evaluation of spermatozoa isolation than single-labeled FISH and quinacrine staining (10). Flow cytometry has also been suggested to be a highly accurate method for sperm separation (7). Although many healthy humans have been born by this method, the risk of cytotoxicity and mutagenic effects of DNA stains and the ultra-laser beam, reduced fertility and negative effects on the rate of blastocyst formation cannot be ruled out completely (11-14). 

Moreover, expensive equipment is required for flow cytometry, which limits its wide usage (5). Polyvinylpyrrolidon (PVP)-coated silica particles (Pecoll^TM^) were withdrawn from the market in 1996 because of the risk of contamination with endotoxins (15). PureSperm® (a silane-coated silica particle preparation (Nidacon, Molndal, Sweden) is now commonly used (16). Many discontinuous gradient techniques such as albumin and PureSperm are used routinely to separate and purify human motile, morphologically normal spermatozoa, although possible chromatin anomalies cannot be directly determined by these techniques (17). 

A negative correlation had been found between sperm chromatin deficiencies and fertilization rates in in-vitro fertilization and intracytoplasmic injection (18, 19). So a simple, rapid method for detecting high-chromatin-quality sperm has important applications in assisted reproduction techniques. In this connection, chromomycin A_3_ (CMA_3_) staining, which characterizes chromatin condensation, has been used for rapid screening in sub fertile men (20). We aimed to determine whether the separation of X- and Y-sperm and screening for sperm quality are affected by simple separation techniques such as albumin and PureSperm, and whether a combination of routine procedures improved the efficacy of X- and Y-spermatozoa separation and DNA chromatin integrity.

## Materials and methods

**Semen samples and experimental design**

Normal semen samples were obtained from 30 normal healthy donors who attended the Shiraz Infertility Center, Iran. The samples were obtained by masturbation after 2-4 days of sexual abstinence, allowed to liquefy at room temperature for up to 30 min and analyzed according to WHO guidelines (21).The ethics committee of Shiraz University of Medical Sciences approved the use of the volunteer's semen for the present study. This investigation was designed as interventional study.

The samples were divided into three groups. In the first group (n=10) X- and Y-sperm were separated with the PureSperm method; in the second group (n=10) the three-layer albumin gradient method was used, and in the third group (n=10) a combination of the PureSperm and three-layer albumin gradient methods was used (alb/ PureSperm). Many studies showed that the X: Y ratio in normal semen does not differ significantly from 1:1, so a control group was not used (12, 13). All samples were centrifuged at room temperature. 

**PureSperm gradient method**

One milliliter of liquefied semen was layered over 2.0 ml 40% over 2.0 ml 80% PureSperm and centrifuged for 20 min at 360 g. The supernatant was discarded and the pellet was diluted with Ham’s F-10 medium (Sigma-Aldrich, Steinheim, Germany) and centrifuged at 360 g for 10 min twice to remove any residual silica particles. After the supernatant was removed, the samples were fixed in methanol/glacial acetic acid (3:1) (Merck, Damstad, Germany) for 30 min, and dropped onto clean slides that were marked with orienting scratches to better identify the sperm fields. They were then air dried and stored at -20ºC.

**Three-layer albumin gradient method**

The method was as described for protocol 3 by Beernink et al (22) with some modifications. 0.5 ml fraction of the washed spermatozoa was then gently placed over 1.0 ml of 10% human serum albumin (HSA) (Sigma) and incubated for 60 min. The upper layer of the spermatozoa was removed and the 10% albumin layer was centrifuged at 450 g for 10 min. The pellet was re-suspended in Ham’s F-10 to a final volume of 0.5 ml and then layered onto 1.0 ml 12.5% HSA over 1.0 ml 20% HSA and incubated. After 30 min, the top layer was removed, and after 60 min the 12.5% layer was removed. The bottom layer (the 20% HSA layer) of the column was diluted with Ham’s F-10 and centrifuged at 360 gr for 10 min. The supernatant was discarded, leaving 1.0 ml. The sperm were fixed in methanol/glacial acetic acid 3:1, dropped onto slides and air dried, and then stored at -20ºC.

**Combined albumin gradient and Pure** **Sperm separation**

Sperm were prepared by the PureSperm procedure and then immediately subjected to three-layer albumin gradient separation as described above.

**Double fluorescence in situ hybridization procedure**

Sperm pretreatment: Dried, frozen sperm samples were decondensed by immersing the slides in 3.0 M NaOH (Sigma-Aldrich) for 3 min, washed with PBS (Sigma), dehydrated through an ethanol series and air dried. The slides were examined by light microscope to verify head condensation changes.

**Probes/slide preparation and assay procedure**

The probes used were X centromere Xp11.1-q11.1 (DXZ1) with spectrum Green® fluorophores for the X chromosome (FITC spectrum) and Y centromere Yp11.1-q11.1 (DYZ3) with spectrum Red® fluorophores for the Y chromosome (Texas red spectrum). Both were purchased from Cytocell (Cambridge, UK). 

In-situ hybridization was done according to the manufacturer’s instructions. In brief, for each slide a mixture of 5.0 µl of each probe was applied on the fields marked on the slides under a coverslip and sealed with rubber cement. The slides which contained the target probes were denatured at 73^o^C for 5 min on a hotplate (Bibby, Staffordshire, UK) and then incubated for at least 6 h or overnight in a humid incubator (Napco 6101, Chicago, IL, USA). Then the slides were washed in 0.4× standard saline citrate solution (SSC) at 73^o^C for 2 min, immersed in 0.05% tween 20 and 2 SSC (Merck) for 30 s, and air-dried in a dark room. Counterstaining was done with 5.0 µl of 4′, 6-diamidino-2-phenylindole in antifade solution (Cytocell).

The sperm nuclei were examined at 1000× magnification under a Nikon E800 Epifluorescence microscope (Nikon, Tokyo, Japan) equipped with a triple-pass filter. In each slide 200-500 spermatozoa were scored by a single observer who was blind to the separation method used. Only spermatozoa with a single tail and oval nuclei were scored. Those with overlapping heads and nuclei were excluded. Sperm with a single green (FITC) or red (Texas red) spot were classified as X- or Y- bearing spermatozoa, respectively. In scoring, we used the criteria of Yan and Huai (2). 

Spermatozoa with a single green or two green signals were classified as X sperm, and those with a single red or two red signals were classified as Y sperm, while those with either green and red signals or lacking any signal were disregarded.

**Chromomycin A**_3_

The slides from three experimental groups were treated with 100 µl of CMA_3_ solution (Sigma) (0.25 mg/ml in McIlvaine buffer, pH7.0, containing 10 mM MgCl_2_) at 4^o^C for 20 min. The slides were rinsed in buffer and mounted with buffered glycerol (1:1). Microscopic analysis of the slides was done by a Nikon Te2000 microscope with the appropriate filters. Sperm with bright green fluorescence were classified as having abnormal chromatin packaging, and those one with dull green fluorescence as having normal chromatin packaging.

**Statistical analysis**

The data were analyzed with one-way analysis of variance (SPSS 11.5) and the Kruskal-Wallis test. P≤0.05 were considered statistically significant.

## Results

**Figure 1:**
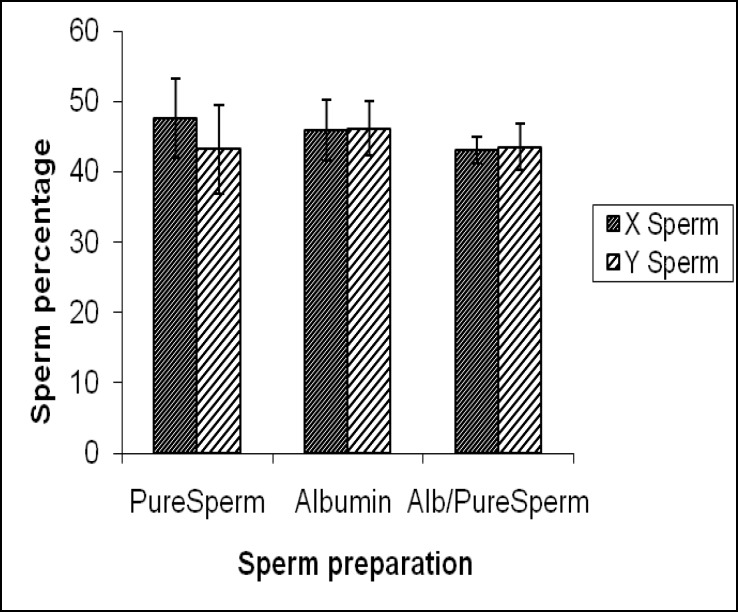
Mean percentage ±SD of X- and Y-bearing spermatozoa after pure sperm®, albumin gradient and combined albumin/pure sperm separation

The effect of the PureSperm, albumin and combined gradient separation methods on the ratio of X- and Y- bearing spermatozoa is summarized in Figure 1. The proportion of haploid X- bearing spermatozoa was slightly higher (4.40%) with PureSperm separation than in putative normal sperm samples (1:1). The overall ratio of X- to Y- bearing spermatozoa with these methods was 47.85±5.67 to 43.19±6.27. The proportion of Y- bearing spermatozoa (46.13±3.83) was slightly higher in albumin gradient-separated fractions, but this difference was not statistically significant (p=0.66). 

**Figure 2:**
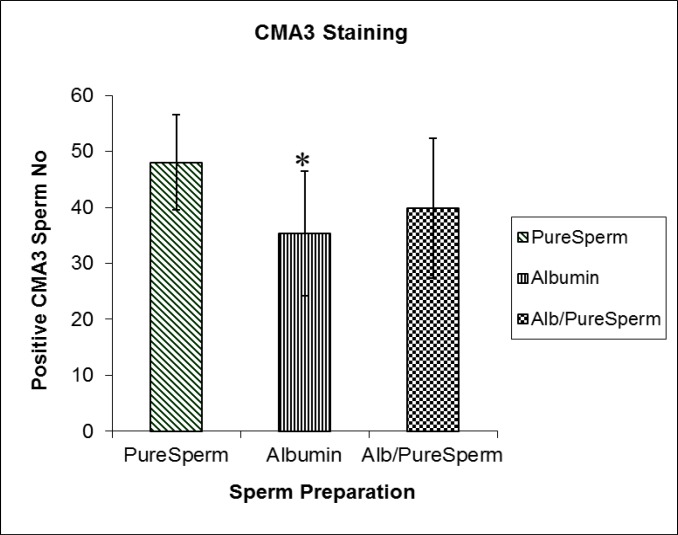
Mean percentage ±SD of CMA_3_-positive spermatozoa after pure sperm®, albumin gradient and combined albumin/pure sperm separation.

The frequency of X- bearing spermatozoa was 45.91±4.36. The results showed no significant difference in the proportion of X- to Y- bearing spermatozoa (p=0.66) with the combined alb/ PureSperm gradient separation method. The overall ratio of X- to Y- bearing spermatozoa was 43.04±1.85 to 43.49±3.27. Statistical analysis showed that there were no significant differences in the counts of X- or Y- bearing spermatozoa among the three methods. In sperm separated with albumin gradients, the mean percentage of spermatozoa positive for CMA_3_ staining was 35.3±8.49 (Figure 2). This percentage was significantly lower than when PureSperm preparation were used (p=0.053). No significant differences were found in the proportion of CMA_3_-positive spermatozoa between the combined alb/ PureSperm method, separation by albumin gradients (p=1.00) or by PureSperm alone (p=0.348). 

*p≤0.05 relative to pure sperm.

## Discussion

The percentage of X-bearing spermatozoa departed slightly from the putative 1:1 ratio when 2-layer discontinuous PureSperm gradient separation was used, although the difference was not statistically significant. Aleahmad *et al* (23) found a higher proportion of X-bearing spermatozoa in the bottom layer than in the top layer of 8-layer discontinuous PureSperm gradients. They claimed that "this procedure was not a reliable method for clinical purposes". There seem to be no further studies of the performance of PureSperm. A slight enrichment in Y-bearing spermatozoa was found by passage of spermatozoa through 3-layer albumin gradients, but again, the difference was not significant. It has been shown that discontinuous albumin gradients do not enrich Y-bearing spermatozoa (9, 24). As in the present study, Aribarg *et al* reported a nonsignificant change in the percentage of Y-sperm from 49.8% before separation to 51% after separation (25).

One earlier study showed that the Y-sperm ratio decreased significantly to 39% in samples processed with Percoll+NycoPrep (26). In the present study, our results with a combination of PureSperm and albumin gradients showed only 0.45% enrichment in Y-bearing spermatozoa. In addition, centrifugation and passage through different layers decreased the sperm count, thus this separation technique may be unsuitable for insemination programs. This variation may be related to applying two different combined gradients.

The mechanism of sperm enrichment is not fully understood. Madrid-Bury *et al* claimed that capacitation (acrosomal reaction/ hyperactivation) plays a more important role than motility in the separation of X- and Y- spermatozoa (27). Another parameter is density differences between X- and Y- spermatozoa, which might be related to their DNA content and sperm mass (28, 29). 

Wolf *et al* believed that if density gradients are prepared in discontinuous fractions and the sperm layered on the top, the spermatozoa penetrate the layers, with the extent of penetration depending on sperm mass and motility. In contrast, with gradient centrifugation, differences in sperm mass are more important than differences in motility (29). 

In the present study, centrifugation with PureSperm gradients led to a slight but nonsignificant enrichment in X-sperm. With albumin gradient centrifugation, we obtained a slight but nonsignificant enrichment of Y-sperm. Various factors may explain the slight differences in the results with different techniques. For example, it was suggested that smaller volumes (1.0-4.0 ml) of the gradients may be insufficient to promote separation (29). In our experiments, we used small volumes of the gradients. It has also been suggested that protein differences between X- and Y-sperm (30) and interactions of both types of sperm with materials in the gradients (31) may influence the results with different mechanisms of enrichment.

In the present study the results of CMA_3_ staining differed significantly (p=0.053) between the three-layer albumin gradient and PureSperm methods for recovering spermatozoa with normal chromatin integrity. However, there's no statistically significant differences in the mean values were found for the albumin and combined alb/PureSperm gradients. 

Sakkas *et al* reported that both Percoll and PureSperm are able to significantly reduce spermatozoa with nuclear anomalies determined by CMA_3 _staining and nick translation assays. PureSperm also appeared to be more efficient than Percoll separation in detecting chromatin and nuclear anomalies (32). Little seems to have been published regarding the effectiveness of albumin gradient separation. We also showed that the proportion of CMA_3 _-positive spermatozoa in combined alb/PureSperm gradient assays was lower than with PureSperm alone, but higher than with albumin gradients alone. 

However, these differences were not significant, suggesting that the passage of spermatozoa through different gradients yielded no advantages in terms of removing abnormal spermatozoa compared to routine gradients such as albumin or PureSperm alone. However, Kheirollahi-Kouhestani *et al* tested Zeta® and PureSperm and reported that these methods improved the quality of semen in terms of chromatin packaging and there's no statistically difference between them (33).

We conclude that routine separation methods might slightly enrich X- or Y-bearing spermatozoa, but this effect was not significant for clinical purposes. In our study the combined alb/ PureSperm method had no advantages for determining the sex ratio or improving chromatin integrity in comparison to the simple gradient separation method. 

## Footnote

***This article was taken from the master’s thesis for the M.Sc. degree.***
